# A Critical Review of the Effectiveness of Biochar Coupled with Arbuscular Mycorrhizal Fungi in Soil Cadmium Immobilization

**DOI:** 10.3390/jof10030182

**Published:** 2024-02-28

**Authors:** Xin Fang, Xinqing Lee, Gratien Twagirayezu, Hongguang Cheng, Hongyu Lu, Shenglan Huang, Linbo Deng, Bo Ji

**Affiliations:** 1 State Key Laboratory of Environmental Geochemistry, Institute of Geochemistry, Chinese Academy of Sciences, Guiyang 550081, China; fangxin21@mails.ucas.ac.cn (X.F.); lee@mail.gyig.ac.cn (X.L.); tgratien0@gmail.com (G.T.); luhongyu21@mails.ucas.ac.cn (H.L.); huangshenglan9666@163.com (S.H.); denglinbo23@mails.ucas.ac.cn (L.D.); richyu1@outlook.com (B.J.); 2 University of Chinese Academy of Sciences, Beijing 100049, China; 3 College of Resources and Environment, Yangtze University, Wuhan 430100, China

**Keywords:** soil, cadmium, contamination, health, remediation, environment

## Abstract

Cadmium-contaminated soil significantly threatens global food security and human health. This scenario gives rise to significant worries regarding widespread environmental pollution. Biochar and arbuscular mycorrhizal fungi (AMF) can effectively immobilize cadmium in the soil in an environmentally friendly way. Existing studies have separately focused on the feasibility of each in remediating polluted soil. However, their association during the remediation of cadmium-polluted soils remains unclear. This review paper aims to elucidate the potential of biochar, in conjunction with AMF, as a strategy to remediate soil contaminated with cadmium. This paper comprehensively analyzes the current understanding of the processes in cadmium immobilization in the soil environment by examining the synergistic interactions between biochar and AMF. Key factors influencing the efficacy of this approach, such as biochar properties, AMF species, and soil conditions, are discussed. The influences of biochar–AMF interactions on plant growth, nutrient uptake, and overall ecosystem health in cadmium-contaminated environments are highlighted. This review indicates that combining biochar and AMF can improve cadmium immobilization. The presence of AMF in the soil can create numerous binding sites on biochar for cadmium ions, effectively immobilizing them in the soil. Insights from this review contribute to a deeper understanding of sustainable and eco-friendly approaches to remediate cadmium-contaminated soils, offering potential applications in agriculture and environmental management.

## 1. Introduction

Soil contaminated with heavy metals has emerged as a critical environmental concern [1,2,3]. Heavy metals are rapidly absorbed by the roots of plants, thereby adversely affecting the physiology and growth of the plant [4]. After translocation through the plant, they may accumulate in the aboveground tissues and enter the human body via the food chain, causing a danger to human health [5]. Currently, cadmium is emerging as a particularly urgent concern due to its widespread occurrence surpassing acceptable levels, especially in China [6]. For instance, based on a 2014 joint study on China soil pollution conducted by the Ministry of Land and Resources (MLR) and Environmental Protection (MEP), cadmium levels exceeded the acceptable threshold of 7%, establishing it as the predominant soil pollutant [7]. This has caused cadmium to be highlighted by the China Environmental Bulletin (2021) as the primary pollutant in agricultural soil [8]. In light of this, there is an urgent need to investigate novel and sustainable remediation approaches to protect ecosystems and human health.

The current approaches for remediating soil cadmium contamination are categorized into physical, chemical and bioremediation methods [9]. The physical method encompasses isolation embedding, soil replacement, and an electrokinetic approach [10]. However, these approaches are expensive and only appropriate for limited and highly contaminated areas, destroying soil structure and causing soil fertility loss [11]. Standard chemical remediation methods include chemical stabilization, solidification/stabilization and soil washing [12,13]. Fixatives and chemical reagents account for a significant portion of the total cost of these procedures. The reaction time of fixatives, crucial in addressing soil contamination, is inherently constrained and this limitation is further compounded by the restrictions imposed by prevailing soil conditions. Heavy metal reactivation and chemical reagent persistence in the soil all represent ecological risks [14]. Therefore, a technique beneficial to the environment, both economic and in situ, such as bioremediation, is highly appreciated.

The substantial advancements in bioremediation have led to its reputation as a sustainable, economical, and ecologically friendly alternative to other remediation approaches [15]. Among them, AMF can form symbiosis with the majority of land plants [16,17]. They can provide nutrients (such as N and P) for host plants, protect hosts from biological stress (such as pathogens), improve the host’s growth environment, and promote host development and growth [18,19,20]. Meanwhile, it is prominent in remediating cadmium-polluted soil [21]. However, its remediation effect on soil cadmium pollution is affected by cadmium concentration, host, and type [22,23,24].

To overcome the limitations of depending solely on AMF for mitigating soil cadmium pollution, the powerful bioremediation agent, biochar, can be synergistically combined with it. Biochar is a carbon-rich material created through the process of biomass pyrolysis. It possesses unique structural and physicochemical characteristics [25,26,27], considerably enhancing soil characteristics [27]. This increases nutrient content and regulates the water-holding capacity of the soil [28,29]. Biochar can also elevate biomass and alleviate stress damage to the plant [27,30]. In addition, it is also widely used as a passivator, which has a noticeable effect on passivating soil cadmium [31]. The tandem use of biochar coupled with AMF could overcome the limitation of using AMF alone to remediate cadmium under certain conditions. The combined application of these two entities holds promise, yet a rigorous examination of their efficacy, influencing factors, and potential ecological implications, is essential to ascertain the reliability and versatility of this remediation approach. In addition, the integrated remediation technology demonstrated synergistic effects in curtailing the migration and transformation of cadmium, mitigating physiological stress in plants, and fostering plant biomass [32]. However, this synergistic effect is ongoing, and their interaction mechanism must be thoroughly elucidated.

This review aims to critically evaluate and synthesize existing knowledge on soil cadmium immobilization through the collaborative action of biochar and AMF. This review also seeks to elucidate the mechanisms underlying the immobilization of cadmium, explore the practicality of this approach across diverse environmental settings, and identify knowledge gaps that warrant further investigation. Ultimately, this review contributes valuable insights into the current status of research on soil cadmium immobilization, offering a foundation for future advancements and sustainable soil remediation strategies.

## 2. Research Methodology

To carry out this study, full-text open-access or requested versions of the relevant English-language publications were used. The inclusion of papers was based on the following criteria: (1) accessibility of full text; (2) presence of information on biochar research; (3) documents offering sustainable applications of biochar for remediation; and (4) peer-reviewed articles written in the English language. Conversely, articles failing to meet the following criteria were excluded from this review: (1) articles written in languages different from English; (2) non-reviewed publications like preprint; (3) papers categorized as the “grey literature”, encompassing school thesis, dissertations, news articles, summaries of scientific events and technical reports; among others. This review paper was also conducted based on the PRISMA 2020 procedures listed by Page et al. [33]. The detailed steps used to obtain articles for this review paper can be found in the Supplementary File, explicitly outlined in Figure S1. Random Forest Analysis was also used to evaluate and understand the complex interactions between biochar, mycorrhizal fungi, and soil cadmium immobilization.

## 3. Geochemical Behavior of Cadmium in the Environment

Typically, cadmium can enter the environment via anthropogenic or natural processes [34]. Naturally, it may occur through atmospheric deposition, including sources such as volcanoes, forest fires, sea spray, and soil particles. Additionally, rock weathering, involving sulfides, carbonates, and phosphorites, can contribute to cadmium release, along with processes like acidification and the existence of organic matter. On the anthropogenic front, human work can augment cadmium in the environment. These include atmospheric deposition from industrial processes, fossil fuel combustion, and agricultural purposes such as using phosphate and nitrogen fertilizer and sewage sludge. Other significant sources involve traffic emissions, groundwater extraction, mining activities, and landfill disposal.

**Figure 1:**
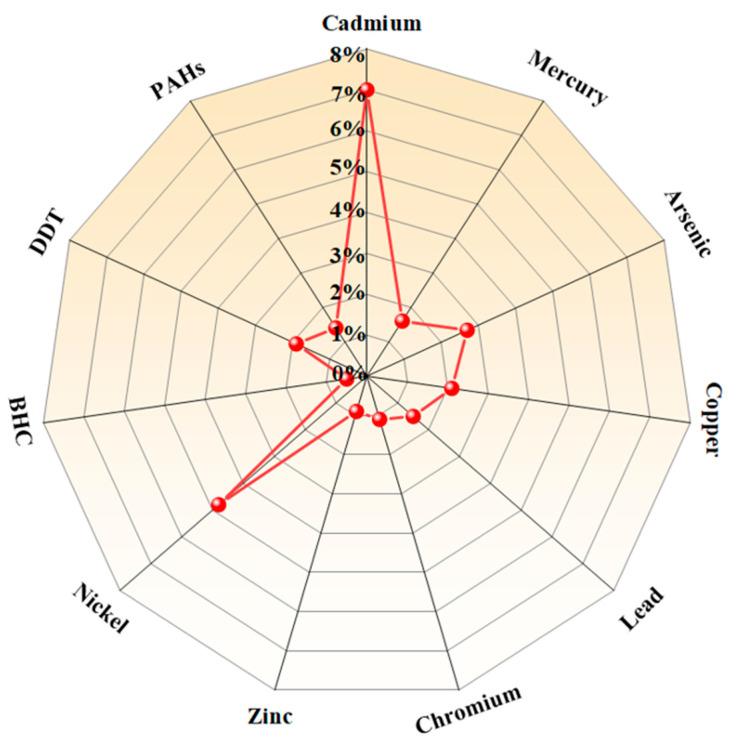
Environmental pollutants in China were assessed in 2014. The acronyms PAHs, DDT and BHC indicate polycyclic aromatic hydrocarbons, dichlorodiphenyltrichloroethane, and benzene hexachloride, respectively.

Typically, cadmium can exhibit mobility through soil and water, particularly under acidic conditions, potentially reaching groundwater [35]. As depicted in Figure 1, cadmium has emerged as the predominant pollutant in the soil environment in China by 2014 [7]. This observation underscores the global significance of the cadmium issue, portraying it as a pervasive concern that extends its regional ramifications. It is well known that elevated cadmium levels in the soil can persist for extended periods, resulting in widespread contamination and potentially affecting other environmental compartments [36]. The chemical form of cadmium has an influence on its bioavailability, with certain forms being more readily absorbed by plants and animals [37]. Typically, the bioavailability of cadmium in the soil environment can vary based on its chemical forms. Different forms of cadmium may exhibit distinct levels of accessibility and uptake by plants or other organisms. This variation in bioavailability is influenced by factors such as the speciation and complexation of cadmium in the soil, which can impact its mobility and potential for absorption by living organisms.

Plants can quickly take up cadmium and accumulate in their tissues [38,39]. This accumulation leads to plant toxicity, affecting its growth and development, reducing crop yield, and impacting the quality of the harvested products [37]. Soil pH and competing ions play a role in determining cadmium bioavailability [40]. Cadmium can disrupt plants’ absorption of vital nutrients, competing with other elements, such as zinc and iron, and leading to nutrient imbalances [41]. This disruption in nutrient availability can further impair plant health and growth. Cadmium can leach into groundwater from contaminated soil, leading to water pollution [34,42]. Cadmium can accumulate in aquatic organisms over time [43], affecting marine ecosystems and human health. Contaminated water sources can also threaten human health if used for drinking or irrigation. Plants absorb cadmium from the soil, and when animals consume these plants, it can accumulate in their tissues [38,43,44]. Bioaccumulation can lead to higher cadmium concentrations in organisms, potentially contaminating humans if they consume contaminated food [45,46]. Cadmium can negatively impact soil microbial communities [40], affecting the overall balance of the soil ecosystem. Cadmium persists in soils, especially in areas with continuous contamination sources. Over time, cadmium accumulation in the soil can have long-lasting effects on soil quality and ecosystem health [37].

Chronic exposure to cadmium through direct contact with contaminated soil or consumption of crops grown in contaminated areas can pose health risks to humans [47]. Cadmium can induce a range of health issues, including but not limited to kidney damage, dysfunction, and neurological disorders. Additionally, it can contribute to lung damage, demineralization of bones, liver damage, reproductive system problems, irritation of the gastrointestinal tract, cardiovascular issues, and respiratory problems, among others. This shows that the widespread occurrence of cadmium, associated with its intricate geochemical behavior, is an environmental concern. Therefore, understanding the geochemical behaviors of cadmium is crucial for assessing its environmental effects and implementing effective management strategies to mitigate its potential harm. In addition, comprehensive attention and collaborative efforts on an international scale are urgently needed.

## 4. Biochar Effects on Cadmium Behavior in Soil–Plant System

**Figure 2:**
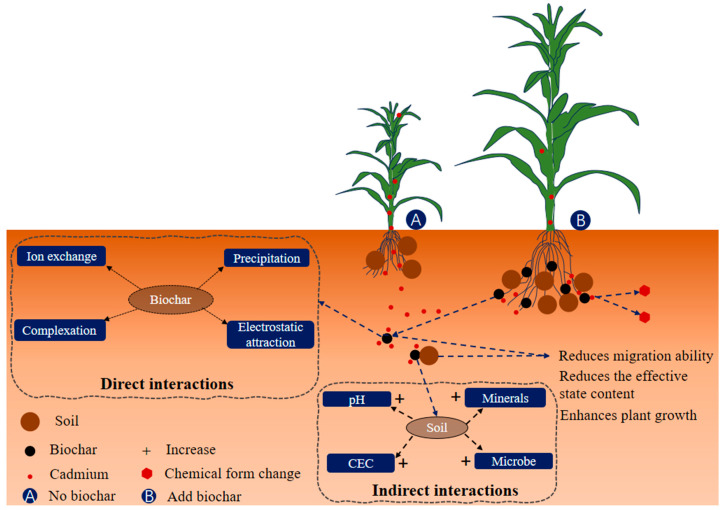
Effect of biochar on cadmium migration and transformation. The labels of CEC represent cation exchange capacity.

Typically, biochar and cadmium interactions in the soil are divided into direct and indirect effects. The immediate results include ion exchange, electrostatic adsorption, mineral precipitation and functional group complexation, among others [48]. The indirect effects encompass alterations in soil physical and chemical attributes, as well as biological properties like pH and the structure of microbial communities. These interactions diminish the movement and bioavailability of cadmium in the soil environment. Simultaneously, they facilitate the transformation of cadmium into stable forms, thereby restraining the absorption and enrichment of cadmium by plants (Figure 2).

### 4.1. Biochar Effects on the Migration of Cadmium in the Soil Environment

Physical and chemical processes like precipitation–dissolution, oxidation–reduction, adsorption–desorption and complexation impact the chemical speciation changes of heavy metals in the soil, causing them to exhibit different migration abilities, closely related to soil environmental conditions [49]. Alterations in soil pH play an important role in modifying the migratory capacity of cadmium within the soil [50]. During pyrolysis, alkali cations present in biomass and alkaline substances like oxides, hydroxides, and carbonates can elevate soil pH and diminish the migration ability of cadmium [51].

In the study carried out by Jia et al. [52], cotton stalk biochar and NaOH-modified cotton stalk biochar were separately added into acid soil at a ratio of 4%, resulting in a respective soil pH increase of 1.32- and 0.87-fold, respectively. The available Cd content in soil decreased by 71% and 57.6%, respectively. The Cd level in the soil pore water decreased by 95% and 87.5%, reducing Cd migration in soil. Han et al. [53] applied wheat straw biochar into the soil at varying ratios (ranging from 0% to 5%), resulting in a notable enhancement of soil pH by a factor of 0.33 to 0.90. This intervention also substantially reduced available cadmium concentration by 2.19% and 15.89%, respectively. These findings indicate significant mitigation of cadmium mobility in the soil due to the applied wheat straw biochar.

Similarly, Meng et al. [54] illustrated that incorporating different types of biochar into the soil at a concentration of 3% led to an approximately 2-fold increase in the pH of both loamy sand and sandy loam soils. The pH elevation, acting as an indirect factor, markedly reduced the available cadmium content in loamy sand soil by 30.6% and in sandy loam by 25.9%. This indicates that a higher soil pH could decrease the migration of cadmium in the soil environment. It was revealed that the pH of manure biochar is generally higher than cellulose biochar [55]. Therefore, adding manure biochar into the soil to control cadmium migration could be preferable.

Biochar can directly immobilize cadmium and fix it in the soil environment. Different types of biochar with varying mass fractions were added to cadmium-contaminated soil. Except for the 0.1% bamboo charcoal treatment, the passivation rate of cadmium by adsorption in other biochar treatments reached more than 30% [56]. By adding rice straw biochar generated at different temperatures, the adsorption capacity of rice straw biochar produced at 700 °C reached 72.57 mg/g through co-precipitation and surface complexation, and the residual cadmium content went up to 26.78%, which stopped cadmium from moving up the soil column [57]. Similarly, compared with the control group, adding biochar in the polluted soil led to a decrease of 32% in the availability of DPTA-extractable cadmium. This decrease in availability signifies a diminished migration ability of cadmium. The mechanism by which biochar adsorbs and fixes cadmium is linked to its abundant aromatic components and active surface functional groups [58]. Ultimately, the direct passivation of cadmium by biochar is another important reason for controlling the migration of cadmium in the soil environment. This review suggests that in exploring the enhancement of biochar’s passivation effect, it is necessary to consider its modifications to increase functional group number, specific surface area, and pore structure [59].

### 4.2. Biochar Effects on Cadmium Speciation in the Soil Environment

Cadmium in the soil can exist in various forms, including exchangeable and carbonate-bound states (extractable by weak acids), reducible forms (associated with iron and manganese oxides), oxidizable configurations (linked to organic matter), and residual states (fixed within primary and secondary minerals) [60]. The exchangeable and carbonate-bound states are the most unstable forms, with solid migration and transformation abilities, high bioavailability, and easy absorption and utilization by organisms. The oxidizable and reducible forms of cadmium are relatively stable, but when the soil environment changes, these forms of cadmium may be transformed into bio-available forms; residual cadmium is considered to be the steadiest form in the soil and is difficult to be absorbed and utilized by organisms.

Under the same conditions, applying different types of biochar can alter the form of cadmium in the soil and promote the transformation of cadmium from an unstable state to a stable state. According to the study of Lu et al. [61], when soil polluted with cadmium was amended with either rice straw or bamboo biochar, the amount of acid-extractable cadmium was reduced by 18.6% and 17.6%, respectively. In comparison, oxidizable cadmium rose by 31.2% and 15.6%, respectively. The effect of biochar derived from rice straw is better than that from bamboo. Biochar derived from chicken manure and green waste increased the proportion of oxidizable and reducible cadmium in the soil [62]. However, green waste biochar decreased the ratio of acid-extractable cadmium by 21.1% and increased the percentage of oxidizable, reducible, and residual cadmium by 15.6%.

According to the study carried out by Zhu et al. [63], the addition of biochar derived from peanut shells and wheat straw in paddy soil considerably lowered the content of acid-extractable cadmium in the soil environment. It significantly increased the oxidizable and residual cadmium content. Concurrently, the addition of peanut shell biochar at a 5% concentration resulted in a decrease in the content of acid-extractable cadmium. This led to a respective 27.76% and 37.87% enhancement in the residual cadmium content, surpassing the effects observed with wheat straw biochar at 21.03% and 24.88%. Biochar mainly changes the form of cadmium in the soil in the following ways: It increases the soil pH so that cadmium precipitates with carbonate, phosphate, and hydroxide ions. Additionally, biochar amplifies the occurrence of alkaline functional groups on the surface of soil colloids, thereby converting cadmium, which it initially extracts through acidic means and converts into various forms of cadmium [64]. Biochar augments soil nutrients like phosphorus and organic matter, resulting in an elevation in the reducible cadmium content [65], and the adsorption of cadmium by biochar itself reduces the content of available cadmium. Biochar improves the living environment of soil microorganisms, thereby increasing the proportion of stable cadmium in the soil environment [66].

### 4.3. Biochar Effects on Cadmium Uptake and Accumulation by Plants

Biochar decreases the mobility and effectiveness of cadmium while reducing its absorption and accumulation by plants. According to a study conducted by Puga et al. [67], adding sugarcane biochar to cadmium-polluted soil at a 5% ratio led to a notable increase in the biomass of both the aboveground and underground parts of the plants. Specifically, there was a 32.7% and 50% increase in biomass in the aboveground and underground parts of the plants compared with the control group. The cadmium content in the aboveground and underground parts was lowered by 18.5% and 19%, respectively, and the content of P and K in the plant increased. However, plant roots increased cadmium absorption while absorbing many nutrients. Similarly, Abbas et al. [68] found that the addition of rice straw biochar into cadmium-contaminated soil at a 5% ratio led to substantial increases in root, stem, ear and grain biomass. The enhancements were remarkable, with growth percentages of 83.8%, 186.5%, 200%, and 106%, respectively, compared with the control group. There was a 47% reduction in the cadmium content in the aboveground part, and the available cadmium content in the soil witnessed a decline of 56%. Adding tobacco straw biochar at a ratio of 2% significantly reduced the cadmium concentration in tobacco at each growth stage, with a decrease of 52.78–82.29%, and considerably increased tobacco biomass at each growth stage. During the rapid growth stage, the most substantial increases in biomass were observed in roots (52.6–151.1%) and stems (157.4–207.3%) [69]. Ren et al. [70] added biochar (1%) from waste peanut shells to different levels of cadmium-polluted soil. Biochar enhances both the physical and chemical properties of the soil, mitigating nutrient loss and facilitating the absorption of nutrients by tobacco plants. Compared with the control group, biochar increased the height, number of leaves, root volume, root length, and whole plant (tobacco) biomass by 3 to 35%. This decreased the amount of cadmium in the parts of tobacco that grew above the ground. The mechanisms through which biochar diminishes the absorption and enrichment of cadmium by plants primarily involve the following three points: (1) biochar diminishes the mobility and availability of cadmium; (2) biochar increases both aboveground and belowground plant biomass, creating a dilution effect; and (3) biochar facilitates the absorption of essential mineral elements by plants, enabling them to compete effectively with cadmium.

## 5. Effects of AMF on Cadmium Behavior in the Soil–Plant Systems

**Figure 3:**
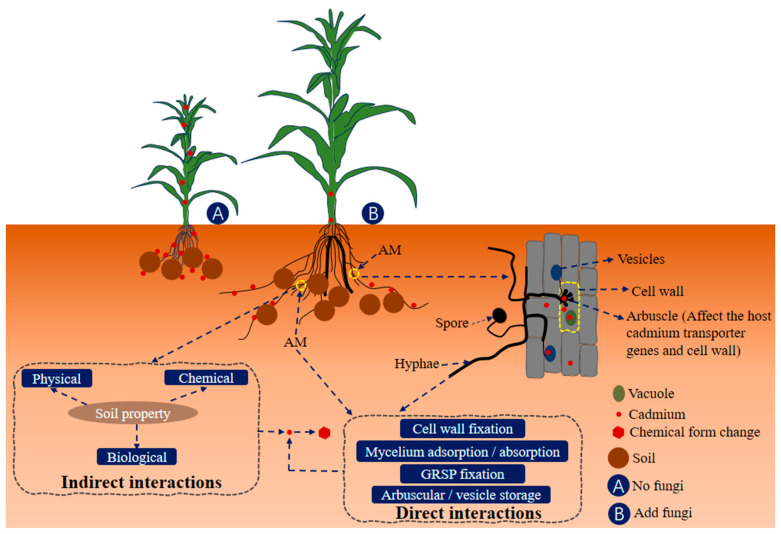
Effects of AMF on the migration and transformation of cadmium. The label of GRSP represents glomalin-related soil protein.

As represented in Figure 3, the processes by which AMF impacts the migration and transformation of the heavy metal cadmium in soil–plant systems can be delineated as follows: (i) It can impact the absorption of cadmium by plant roots through its influence on plant physiology, development and growth; (ii) AMF produces secretions that can change the physicochemical, and biological properties of the soil, changing the chemical form of cadmium in the soil; (iii) the retention, absorption, or adsorption of cadmium by mycorrhizal structure or hyphae; and (iv) AMF regulates the transportation and dispersion of cadmium within the plant [71].

### 5.1. Effects of AMF on Cadmium Behavior in the Soil Environment

The inoculation of AMF can alter the physicochemical properties of soil and the chemical form of cadmium, either directly or indirectly. Based on the study conducted by Wang et al. [72], the inoculation of AMF into the soil resulted in a 29.79% reduction in cadmium content. Additionally, the inoculation of AMF facilitated the transformation of cadmium into a more stable form in the soil. Compared with non-inoculated conditions with the same cadmium concentration, there were higher percentages and amounts of oxidizable, reducible, and residual cadmium in the soil with AMF inoculation across different cadmium concentrations. In pots planted with maize plants, soil inoculated with AMF exhibited a significant decrease in acid-extractable and reduced cadmium concentrations, while the oxidizable and residual cadmium concentrations increased [32]. In the pots planted with alfalfa, the inoculation of AMF significantly decreased the proportion of exchangeable and carbonate-bound cadmium, increased the proportion of residual cadmium by 20.29%, and reduced the proportion of iron-manganese-bound and organic-bound cadmium by 4.61% and 5.95%, respectively [73]. AMF can absorb PO_4_^3−^ and NO_3_^−^ and other anions by extracellular hyphae, increasing the number of OH ^−^ ions in the soil environment and improving soil pH [74]. This can indirectly change the form of cadmium and promote the transformation of available cadmium to stable cadmium by affecting the community structure of rhizosphere microorganisms [75].

In addition, glomalin-related soil protein (GRSP), as a ubiquitous AMF secretion, can directly bind to cadmium and change the chemical form of cadmium in soil. Chen et al. [75] revealed that the inoculation of AMF substantially increased the content of total glomalin (T-GRSP) and easily extractable glomalin (EE-GRSP) in the soil. Compared with the non-inoculated treatment, the EE-GRSP content in the inoculated treatment increased substantially by 116.1%, 103.0%, and 106.0%. As the soil cadmium concentration increased, the content of EE-GRSP in the soil also increased. The combination of glomalin and cadmium lowered the content of available cadmium in the soil, and the effect was more evident in 50 mg/kg cadmium-treated soil. Glomalin was extracted from cadmium-contaminated soil. The findings revealed that the cadmium content in glomalin was 0.02–0.08 mg/g [76]. Gerami et al. [77] demonstrated that the content of T-GRSP and EE-GRSP decreased with an increase in the soil cadmium concentration. Under different cadmium concentrations, the amount of glomalin in *Funneliformis mosseae* (Fm) decreased by 18%, 34%, and 50%, respectively. However, the amount of glomalin made by FM and *Claroideoglomus etunicatum* (Ce) was higher than those of the control and *Rhizophagus intraradices* (Ri). The complexation of T-GRSP and EE-GRSP to Cd was also higher than that of RI fungi. These results could be attributed to the higher strength of FM and CE in terms of cadmium resistance, response sensitivity, and the colonization rate compared with RI. GRSP can effectively bind to cadmium, change its chemical form, and reduce its effectiveness. However, exploring the specific mechanism of GRSP binding to cadmium is still necessary to better understand whether it can be rereleased into the soil environment. To conduct reasonable regulation in the practical application can reduce the risk of cadmium entering the soil again.

### 5.2. Effects of AMF on the Absorption and Accumulation of Cadmium in the Plants

In the soil–plant system, the extracellular hyphae cell wall of AMF blocks the migration of cadmium to plants. Joner et al. [78] showed that AMF mycelium can rapidly adsorb Cd by passive adsorption, and the adsorption capacity of *Glomus mosseae* mycelium to Cd reached 0.5 mg/g. The presence of Cd was detected on the cell wall of AMF mycelium by synchrotron radiation X-ray fluorescence imaging after the mycelium and spores were treated with cadmium [79]. Energy dispersive X-ray spectroscopy also found Cd on the cell walls of the two strains [80]. The adsorption of cadmium on the mycelial wall was related to the components of the mycelial wall, like chitin and melanin, which had binding solid abilities to cadmium [81]. In addition to the direct adsorption of cadmium by the mycelium wall outside the root, the mycorrhizal structure can absorb cadmium in the soil and transport or retain it. The results of compartmental experiments showed that 24% of cadmium in soybeans and 41% of cadmium in corn were from the absorption of the mycelium outside the root from the external compartment [82].

Similarly, Hutchinson et al. [83] revealed that the cadmium absorbed by the mycelium outside the root accounted for 10% of the cadmium absorbed by the plant. By increasing the cadmium concentration (1, 10, and 100 mg/kg), the cadmium content of mycorrhizal plants increased continuously; extraradical hyphae absorbed cadmium from the soil and transported it to the plants [84]. Cadmium absorbed by AMF extracellular hyphae is sometimes not transported to the plants but to the mycorrhizal structure for storage. Synchrotron radiation μX-ray fluorescence was used to assess cadmium in mycorrhizal structures. It was evident that cadmium absorbed by extraradical hyphae was transported to the mycorrhizal structure, and a large amount of cadmium was found in the arbuscular [85]. X-ray fluorescence analysis also found more cadmium in the mycorrhizal structure (vesicles and arbuscule) [79]. In addition, the transport of cadmium by transporters to the AMF vacuole for accumulation may also reduce the accumulation of cadmium in the aboveground part. The adsorption/absorption of cadmium by hyphae, vacuole storage, and retention of AMF structure are essential ways to prevent cadmium from entering plants. Therefore, promoting the formation of a symbiotic structure and AMF growth is helpful to achieve the above process.

The effect of AMF on host plants is another crucial reason affecting cadmium migration. Plant cell walls have a specific retention effect on cadmium. In the soil contaminated with cadmium, the inoculation led to a notable increase in the total sugar content within the pectin and HC1 (hemicellulose 1) of the host cell wall, with enhancements of 1.13 times and 1.11 times, respectively, compared with the non-inoculated treatment. The aldehyde acid content was raised by 1.19 times and 1.23 times; the content of lignin was increased by 32.3%; and the activity of L-phenylalanine ammonia-lyase (PAL) and pectin methylesterase (PME) was increased. The hydroxyl and carboxyl groups number on the root cell wall, making a large amount of cadmium fixed on the root cell wall and preventing the further upward transport of cadmium. The change in cell wall composition by AMF is further amplified based on the modification of cell wall composition induced by cadmium stress, and its mechanism remains to be further explored [86]. AMF indirectly changes cadmium transport by regulating the expression of transport genes in plants. Compared with the control, Fm and Ri substantially decreased the cadmium concentration in roots by 18% and 38%, respectively. One reason why the concentration of cadmium in Ri is lower than that in Fm is that the expression of Nramp5 (encoding Nramp5 transporter) and HMA3 (encoding HMA3 enzyme) genes in Ri treatment is more inferior in roots. In contrast, the expression in Fm treatment is higher. Nramp5 can transfer cadmium from the outside to the root cells. HMA3 can transfer cadmium to vacuoles [87]. In another study, Ri and Fm treatments reduced the expression of OsNramp5 and OsHMA3 genes in rice roots under a low cadmium stress (2 mg/kg) and high cadmium concentration (10 mg/kg) compared with the non-inoculated treatment; the Ri treatment reduced OsNramp5 expression and increased OsHMA3 expression. The FM treatment reduced OsNramp5 expression and did not substantially affect OsHMA3 expression, but the inoculated treatment decreased cadmium concentrations in rice [88]. It can be shown from the above that the regulation of AMF on plant root transport genes needs to be more consistent. The expression of these genes affects the absorption and enrichment of cadmium. To comprehend how AMF impacts cadmium absorption and enrichment at the molecular level, it is helpful to learn more about how they control the expression of host plant genes.

In addition, when cadmium enters the plant, AMF changes its distribution and morphology [89]. The study conducted by Zhang et al. [43] indicated that inoculation led to more than 90% of cadmium and soluble components of maize in the cell wall, predominantly in vacuoles. This resulted in a reduced inorganic and water-soluble cadmium content in the plants associated with increased pectin and protein-bound cadmium. Moreover, the transformation of cadmium into an inactive form occurred, significantly limiting its migration within plants. These effects were also correlated with increased levels of glutathione and phytochelatin. Li et al. [90] indicated that the inoculation of AMF enhanced the proportion of pectin, protein-bound cadmium, and insoluble phosphate cadmium in the stems and roots of rice and lowered the concentration and balance of inorganic and water-soluble cadmium. Cadmium is mainly retained in the cell walls and vacuoles. Similarly, Wang et al. [91] demonstrated that the inoculated treatment significantly enhanced the binding of cadmium to fruit acid esters and proteins. Simultaneously, it decreased the proportion of cadmium associated with the cell membrane and organelles of alfalfa while elevating the proportion bound to the cell wall. This treatment also resulted in a reduction in the migration of cadmium to the aboveground parts. From the above studies, AMF effectively changed cadmium’s morphology and subcellular distribution in different host plants and reduced the physiological toxicity and migration of cadmium to plants. However, the specific mechanism by which AMF regulates the distribution and morphology of cadmium in host plants needs further exploration. This exploration is essential to better understand how AMF can interact with the host.

### 5.3. Effects of AMF on Cadmium Dynamics in the Soil Environment

Heavy metals continue to impact the survival and reproduction of AMF as an organism. High concentrations of heavy metals are toxic to AMF, making it challenging to complete colonization or recurrence [92]. Five different sources of *G. mosseae* were added to soils with varying concentrations of cadmium (0, 50, and 100 mg/kg). With the increase in cadmium concentration and plant cultivation time, the colonization rate and arbuscular abundance of *G. mosseae* (BEG12) decreased, and the cadmium content of host plant stems was also higher [93]. On the other hand, Gai et al. [22] studied the effect of Cd in soil on the development of AMF under the same host conditions. His study indicated that the total extraradical hyphae (ERM) length and active ERM length of AMF was inhibited under high Cd concentrations (25, 50 mg/kg). Other studies have also found that cadmium inhibits the mycelial growth of AMF and spore germination [94]. Additionally, the content of available phosphorus in the soil environment is a major environmental factor affecting the formation and function of mycorrhiza. A low phosphorus content is conducive to colonizing AMF and constructing a mutually beneficial symbiotic relationship between AMF and host plants. High-phosphorus soil can limit the colonization of AMF and make AMF and host more inclined to form a symbiotic and parasitic relationship [95,96,97].

Taken altogether, abiotic factors affect the germination of AMF spores, mycelial growth, and the formation of mycorrhizal structures [98]. In addition, they can also be impacted by biological factors like the predation of AMF by other organisms and the selection of AMF by host plants [99], which significantly limits the inhibitory effect of AMF on cadmium. Therefore, when AMF is used to control cadmium, other repair methods can be used to improve the living environment of AMF and ensure its symbiotic relationship with host plants.

## 6. Effects of Biochar Coupled with AMF on Cadmium Behavior in Soil–Plant Systems

An ideal remediation method is to increase plant biomass while minimizing the migration and enrichment of cadmium. Following this principle, the results of this research in this direction are categorized into synergistic and non-synergistic effects. As shown in Table 1, the synergistic influences indicate that the combined impact is more substantial than using the elements individually, resulting in reduced plant cadmium enrichment, alleviation of plant physiological stress, and decreased soil cadmium availability and migration. Conversely, the non-synergistic impact suggests that the combined effect is not significantly more potent than using the elements individually, or the result may not be pronounced in reducing plant cadmium enrichment, alleviating plant physiological stress, and decreasing soil cadmium availability and migration.

### 6.1. Effects of Biochar Associated with AMF on the Migration and Transformation of Cadmium in Soil

The combined use of biochar and AMF showed a synergistic effect on reducing the migration and transformation of cadmium. The study carried out by Liu et al. [13] revealed that in the soil contaminated with 3 mg/kg of cadmium, the combined use of biochar and AMF resulted in a 51.99% reduction in acid-extractable cadmium and a 41.31% reduction in reducible cadmium. Simultaneously, there was a notable increase of 337.3% in oxidized cadmium and 77.83% in residual cadmium. At 6 mg/kg, the acid-extractable and reducible cadmium lowered by 47.57% and 31.93%, respectively, and the oxidized and residual cadmium increased by 131.7% and 77.8%, respectively. The findings from the study conducted by Qiu et al. [107] indicated that the combined use reduced the proportion of acid-extractable cadmium to 45%, and residual cadmium increased from 7.14% of the control to 15.61%. The cadmium leaching was determined to be 0.65 mg/kg by calcium chloride extraction, significantly reducing cadmium availability in the soil environment [108].

### 6.2. Effects of Biochar Associated with AMF on Plant Growth and Physiology

Their combined use can enhance growth and physiological aspects under varying concentrations of cadmium-induced stress. In a study conducted by Alotibi et al. [109], soil contained 3 mg/kg of cadmium; the combined treatment resulted in a 15.6% elevation in plant height and a 53.84% increase in stem dry weight compared with the control. Likewise, in the soil with an elevated cadmium concentration of 5 mg/kg, the combined treatment led to a 28.20% rise in plant height and a 48.78% increase in stem dry weight compared with the control. In the soil with a cadmium concentration of 4 mg/kg, the combined treatment demonstrated a substantial increase in the aboveground biomass, underground biomass, and plant height of mulberry compared with the control. Specifically, enhancements were 75.51%, 62.5%, and 43.28%, respectively [110]. In soil with a cadmium concentration of 6 mg/kg, a significant rise in the average fresh weight and plant height of maize was observed, showing increases of 70% and 31%, respectively [110].

In a study conducted by Guo and Li. [110] on plant physiology, it was revealed that the combined treatment significantly decreased the malondialdehyde content across various cadmium levels by 69.1% while simultaneously boosting the antioxidant enzyme activity by 54.3%, 83.4%, and 52%, respectively. Furthermore, the photosynthetic gas exchange parameters of leaves exhibited noteworthy improvements, increased by 23.94%, 31.98%, and 30%, with a simultaneous reduction in intercellular carbon dioxide concentration by 23.4% compared with the control group. The content of photosynthetic pigments increased by 12.9%, 31.48%, and 21.65%, respectively. The study conducted by Alotibi et al. [109] was similar to the above results. Further research also found that the combined use increased plant glutathione by 16.25% and 15.89%, respectively. Lipid peroxidation levels were reduced by 28.85% and 57.1%, respectively. In addition, Ascorbate peroxidase, the amino acid content, glutathione reductase, the glycine betaine content, and total phenols were decreased. The photochemical quenching was reduced by 20.3%, the electron transport rate was decreased by 30.22%, and the non-photochemical quenching was reduced by 34.67%.

### 6.3. Effects of Biochar Associated with AMF on Cadmium Enrichment Reduction in Plant

According to the results reported by Liu et al. [13], the impact of utilizing phytoremediation techniques on reducing the cadmium enrichment is apparent. Additional research has demonstrated that in soil containing 3 mg/kg of cadmium, the underground parts of the plant exhibited a reduction in cadmium content by 42.23%. In contrast, the aboveground parts decreased from 63.67% to 76.41%. Similarly, in the soil with 6 mg/kg cadmium, there was a reduction of 50.06% in the underground parts and a decrease ranging from 67.9% to 76.19% in the aboveground parts. The bioconcentration factor (BCF) and the translocation factor (TF) were decreased by 53% and 28%, respectively. According to the research carried out by Zhao et al. [111], the combined use of specific methods resulted in a substantial decrease in cadmium concentration. Specifically, compared with the control group, the combined treatment led to an 86.4% reduction in cadmium concentration in the underground parts and a 50.5% reduction in the aboveground parts. BCF also showed a significant decrease.

Consistent with these findings, Pu et al. [108] observed a substantial reduction in cadmium concentration in lettuce, with stems and leaves by up to 85% and 89%, respectively. In Chinese cabbage, stems and leaves were decreased by 62% and 56%, respectively. Moreover, another study by Qiu et al. [107] revealed a decline in the BCF and TF. As indicated in the study of Zhuo et al. [112], the synergistic usage of biochar and three distinct arbuscular fungi types exhibited notable efficacy in reducing cadmium content in maize shoots. In the same study, the reductions were 50%, 80%, and 51% for the three fungal types. Furthermore, in the underground and aboveground parts of maize, there were substantial decreases of 89%, 87%, and 88.5%, respectively, indicating a significant mitigation of cadmium accumulation.

### 6.4. Mechanisms of Biochar Combined with AMF on the Migration and Transformation of Cadmium

**Figure 4:**
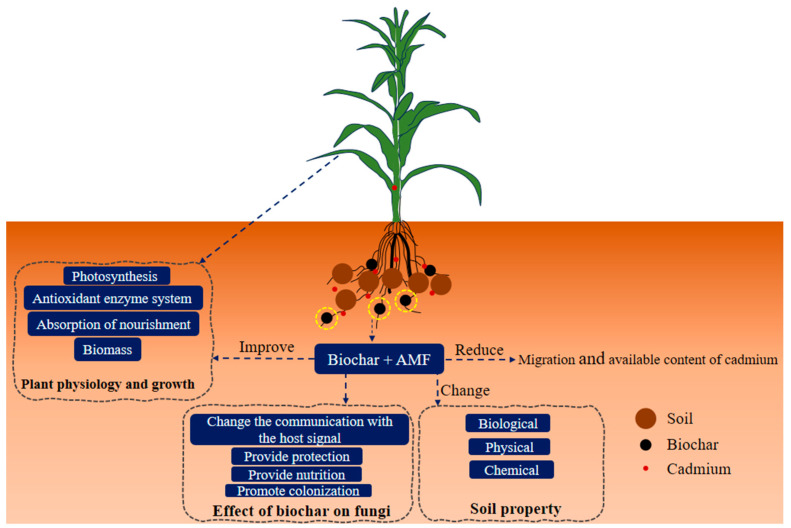
Mechanisms of biochar combined with AMF on the migration and transformation of cadmium.

As depicted in Figure 4, the combined use of biochar and AMF demonstrated a synergistic effect, reducing plant cadmium accumulation, alleviating plant physiological stress, and decreasing the availability and mobility of soil cadmium. It is essential to note that biochar and AMF play distinct roles in this process, and they interact. The effects of the synergistic impact and the effects produced when used alone can be divided into three situations: combined > biochar > AMF, combined > AMF > biochar, or combined > biochar = AMF (both have a poor effect when used alone).

During the synergistic process, biochar assumes a crucial function in fostering the growth and development of AMF, ensuring the standard functionality of mycorrhiza. Warnock et al. [113] suggested four possible mechanisms to explain the effects of biochar on the abundance and function of AMF: (i) changing the physical and chemical properties of the soil, (ii) indirectly affecting AMF by influencing other microorganisms, (iii) altering the process of signal exchange between AMF and plants, and (iv) protecting fungi. Previous studies have shown that elevated levels of heavy metal pollution adversely affect the germination of AMF spores, the development of extracellular mycelia, and mycorrhiza colonization [114]. However, in most studies, the incorporation of biochar has been observed to significantly enhance the colonization of AMF, with the highest colonization rate reaching 92.3% [111]. One reason is that biochar can passivate cadmium in the soil, reducing its toxicity to AMF and protecting the fungi. Moreover, biochar can impede the proliferation of specific soil microorganisms. Qiu et al. [107] showed that the combined use of biochar and AMF resulted in the modifications of the soil microbial community composition, the prevalence of soil microorganisms, and β diversity. It also promoted the growth of microorganisms closely associated with arbuscular mycorrhiza, ultimately enhancing the dominant position of AMF in the soil environment.

In the process of synergistic effect, biochar has the most significant influence on the soil pH and has a more substantial impact on alkalizing soil and fixing cadmium. At the same time, because of the alteration in the soil physicochemical properties and the introduction of nutrients through biochar, there is a promotion of plant growth and nutrient absorption by AMF [115]. AMF can also absorb nutrients through small hyphae within biochar pores and transport them to host plants, promoting plant growth and reducing dilution [116]. Mineral nutrients compete with cadmium when entering plants, reducing the chance of cadmium entering plants [117]. When AMF is in a robust growth state, it retains cadmium through the symbiotic structure [105]. This hinders the migration of cadmium to the plant or its aboveground parts, consequently diminishing the toxicity of cadmium to plants. This regulatory effect is manifested through the modulation of photosynthetic characteristics and antioxidant enzyme activities in the plants [118].

### 6.5. Non-Synergistic Effects of Biochar Combined with AMF in the Soil–Plant Systems

Even if the simultaneous application of biochar and AMF can yield a synergistic effect, this outcome is only uniformly observed in some cases of their combined use, introducing an element of uncertainty. Qiao et al. [106] revealed that using biochar prepared at different temperatures (300 °C, 500 °C) and AMF did not effectively reduce cadmium availability in the soil or increase plant height. Furthermore, there was no discernible interaction between them.

Vejvodová et al. [119] also demonstrated that the addition of different proportions of olive residue biochar (300 °C, 500 °C) combined with AMF in contaminated soil with varying concentrations (high, medium, low) of compounded heavy metals did not effectively increase plant biomass nor reduce the concentration of cadmium in the plants. This ineffectiveness was attributed to the substantial constraints imposed by combined pollution on the development and growth of AMF, possibly influenced by the type and application ratio of biochar. Conversely, the high content of available phosphorus in the soil may also be crucial. It has been demonstrated that an increased phosphorus concentration hinders root infection and reduces the activity of AMF [120,121].

**Figure 5:**
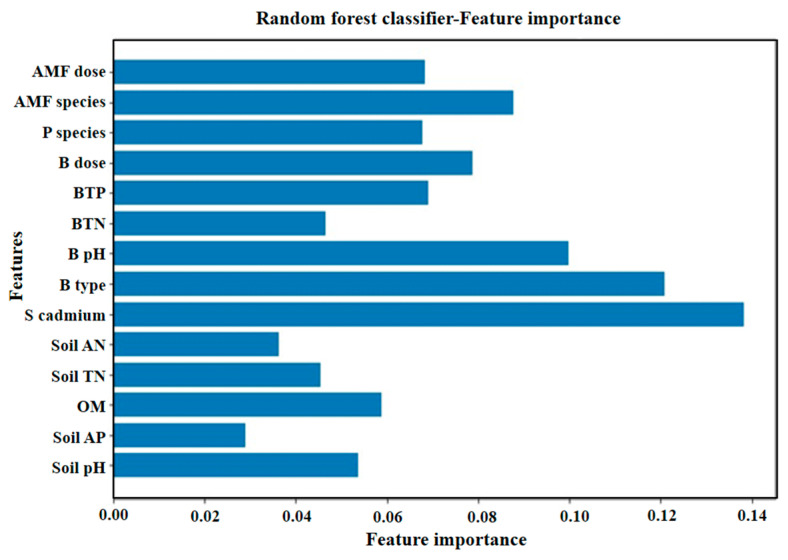
The significance of the characteristics associated with each experimental factor. The letter ‘S’ at the beginning represents the soil index, while the letter ‘B’ indicates the biochar index. ‘AN’ stands for available nitrogen, ‘AP’ for available phosphorus, TP for total phosphorus and ‘TN’ for total nitrogen. ‘B type’ denotes the type of biochar, ‘dose’ refers to the addition amount, and ‘species’ represents the various plant species. ‘P species’ signifies the plant type, and ‘OM’ stands for soil organic matter.

In the other studies, using biochar and AMF has increased the plant nutrient uptake and enhanced plant biomass under low cadmium concentrations. However, its effectiveness in reducing cadmium concentration in plants and cadmium availability in soil is less pronounced than when used individually [122,123]. In some other work, combined use under low concentrations of cadmium conditions can increase plant nutrient uptake and biomass. Still, more effectiveness is needed to reduce cadmium concentration in plants and cadmium availability in the soil environment. The non-synergistic effect of the combined use makes it difficult to use this technology to control cadmium pollution. Indeed, the technology can have a synergistic effect when used. To enhance the comprehension of the factors influencing the synergistic effect of using biochar–AMF, it is crucial to consider additional information presented in Table 2. Random forest analysis shows that biochar type, pH, AMF type, and soil cadmium concentration are the four factors that have the most significant impact (Figure 5). Among them, soil cadmium concentration is the most influential factor. Therefore, a comprehensive consideration is needed to use better technology.

## 7. The Synergistic Influences of Biochar Coupled with AMF on Cadmium Immobilization in the Soil

The prospective effectiveness of the biochar associated with AMF in immobilizing cadmium is promising, although its success depends on a range of factors, some of which enhance and others inhibit its performance. To ensure optimal outcomes, it is imperative to consider site-specific considerations, make judicious choices in selecting suitable biochar and AMF strains, and cultivate a profound understanding of the prevailing soil and environmental conditions. Customizing the application of biochar and AMF to match the distinct characteristics of each site is essential [100]. Factors like soil composition, pH levels, and the current microbial community can considerably influence the effectiveness of cadmium immobilization [75]. The inherent properties of biochar, originating from diverse feedstocks, can vary and impact its capacity to effectively sequester cadmium [125,126]. Likewise, different AMF strains exhibit varying efficiency in nutrient uptake and metal immobilization [88,127].

The synergistic effects between biochar and AMF can be optimized by carefully considering application rates and methods [90]. The implementation of this approach needs a holistic understanding of the specific challenges posed by cadmium contamination in a given location. In addition, the success of this strategy is contingent upon ongoing research efforts to validate its effectiveness based on AMF and the inherent properties of biochar derived from various raw materials and their impact on effective cadmium sequestration, along with their implications in the cadmium sequestration process. Therefore, continuing research and experimentation can uncover the intricacies of the biochar–AMF interaction, providing valuable insights into how this innovative approach can be refined to address cadmium immobilization effectively across diverse environment compartments.

## 8. Recommendations and Prospect

The promising approach of immobilizing soil cadmium through the synergistic application of biochar and AMF has gained attention in environmental research [111]. This review thoroughly assesses the existing knowledge in this domain, providing insights into the effectiveness of biochar–AMF combinations in addressing cadmium contamination in soil. As concerns about soil pollution intensify, understanding the mechanisms behind this remediation strategy is crucial for developing sustainable and practical solutions. Subsequent research efforts should optimize biochar–AMF combinations, considering factors like the optimal ratio, variations in the soil properties, and potential interactions with other soil contaminants.

Long-term field studies should be crucial in assessing the practical applicability and sustainability of this approach across diverse agricultural areas. Moreover, investigations into the influence of climate change on the efficacy of cadmium immobilization using biochar and AMF are imperative. Fluctuations in temperature, precipitation patterns, and overall climatic conditions can influence the interactions within the soil–plant system. This underscores the need to comprehensively understand their dynamics under different environmental scenarios. Future perspectives should also explore the potential impacts on the soil microbial communities and overall ecosystem health. Understanding the broader ecological consequences of employing biochar and AMF for cadmium immobilization is essential to developing environmentally sustainable agricultural practices.

Exploring the mechanisms of the impacts of different combinations involving biochar types, AMF species, and soil cadmium concentrations on the collective results needs to be elucidated in future experiments using a deep learning approach, such as random forest analysis, etc. The biochar type, biochar pH, AMF type, and soil cadmium concentration should be considered before remediation. Identifying the optimal combination for cadmium control based on local conditions necessitates a thorough comprehension of the interactions among different factors and their cumulative influence on the overall effectiveness. To explore the distribution and morphology of cadmium within mycorrhizal structures (hyphae, vesicles, arbuscule, and vacuoles) and subcellular compartments of plants, it is crucial to grasp the pathways through which cadmium moves from the soil to the root surface and vice versa. Understanding its presence, forms, and migration mechanisms in each path is essential for effective cadmium management. This knowledge facilitates the implementation of precise control measures to resist cadmium contamination.

We delved into the interaction mechanism between biochar and AMF, explicitly examining their influence on the structure of rhizosphere microorganisms in plants and the expression of cadmium-related transport genes in roots, is needed. Rhizosphere microorganisms that play a crucial role in shaping the migration and transformation of cadmium, along with the development of mycorrhizal structures and their effectiveness in cadmium control need investigation. Furthermore, the expression of cadmium-related transport genes in roots is vital in determining the ability of cadmium to permeate plants. The utilization of biochar and AMF for cadmium control is currently being investigated through greenhouse pot experiments, and its effectiveness in cadmium containment requires validation through field experiments. Furthermore, the passivation ability of biochar against cadmium diminishes with aging, posing the risk of potential release into the environment. Concurrently, biochar significantly influences the growth and mycorrhizal formation of AMF, consequently affecting the overall efficacy of their combined application. Therefore, the necessity for long-term experiments to substantiate these findings is evident.

## 9. Conclusions

This review underscores the potential synergistic effects of biochar and AMF to immobilize soil cadmium. The combined application of biochar and AMF has demonstrated promising results in reducing cadmium bioavailability, thereby minimizing its negative impact on plants and the environment. In addition, the combined application yields a synergistic effect, resulting in superior outcomes and presenting a novel avenue for ensuring agricultural production and food chain safety. The collective influence of biochar and AMF depends on diverse factors, encompassing biochar type, pH, application quantity, plant species, and soil cadmium concentration. The explained mechanisms, such as biochar’s adsorption capabilities and AMF’s enhancement of nutrient uptake, underscore the multifaceted advantages of this integrated approach. The association of biochar and AMF can contribute to the progression of sustainable soil management practices and play a crucial role in alleviating the environmental and health risks linked to cadmium contamination.

## Figures and Tables

**Table 1:** Positive and negative influences of biochar associated with AMF on cadmium immobilization.

	Influences	Remark	Reference
Positive	Reduced bioavailability	Biochar has a high surface area and can adsorb cadmium, reducing its bioavailability. When combined with AMF, the mycorrhizal network can facilitate the transport of biochar particles and improve their distribution in the soil, enhancing the immobilization of cadmium.	[100]
	Enhanced sorption	The porous structure of biochar provides numerous binding sites for cadmium ions, immobilizing them in the soil. The presence of AMF could increase the efficiency of this process by promoting a more extensive root system and facilitating the interaction between biochar and the rhizosphere.	[101]
	pH modification	Some biochars have alkaline properties that can influence soil pH. An increase in pH can lead to the precipitation of less soluble forms of cadmium, reducing mobility. The mycorrhizal association may further contribute to pH modification in the rhizosphere, enhancing the immobilization of cadmium.	[85,102]
	Mycoextraction	Certain AMF species can accumulate cadmium within their mycelium through mycoextraction. This sequestration reduces cadmium availability in the soil, preventing plant uptake and minimizing the transfer risk to the food chain.	[103,104]
	Microbial community enhancement	Both biochar and AMF can positively influence soil microbial communities. Beneficial microbes can contribute to processes immobilizing cadmium, such as microbial-mediated precipitation and complexation reactions. Biochar may provide a supportive habitat for these microbes, and AMF can further enhance their activity.	[40]
	Improved soil structure	Combining biochar and AMF may contribute to soil aggregation, improving soil structure. Enhanced soil structure can limit the leaching of cadmium, keeping it bound within the soil matrix and reducing the risk of groundwater contamination.	[100]
Negative	Variable effects	The effectiveness of biochar and AMF in cadmium immobilization can vary depending on factors such as biochar type, AMF species, soil properties, and environmental conditions. In some cases, the synergistic effects may be less pronounced, leading to less effective cadmium immobilization.	[32]
	Biochar–AMF characteristics	The success of cadmium immobilization in biochar–AMF may depend on the properties of the biochar used. Some biochars may have limited cadmium adsorption capacity, and their effectiveness could be influenced by feedstock and pyrolysis conditions.	[32]
	Site-specific considerations	The efficacy of the combination may be influenced by site-specific conditions. Soil characteristics, existing cadmium concentrations, and other contaminants can affect the overall success of the biochar–AMF strategy for cadmium immobilization.	[105]
	Long-term stability	The long-term stability of biochar and the sustainability of the mycorrhizal association may influence the persistence of the positive effects on cadmium immobilization. Changes in environmental conditions over time could impact the success of the strategy.	[106]

**Table 2:** Effects of different experimental factors on the use of biochar combined with AMF—represents the different experimental conditions in the same literature; B represents biochar; AN is available nitrogen; AP is available phosphorus; TN is total nitrogen; TP is total phosphorus; Y indicates a synergistic effect, and N indicates no synergistic effect; AMF species refers to the type or use of AMF.

Soil pH	Soil AP (mg/kg)	Soil Organic Matter (g/kg)	Soil TN (g/kg)	Soil AN (mg/kg)	Soil Cadmium Concentration (mg/kg)	Biochar Types	B pH	B TN(g/kg)	BTP(g/kg)	Biochar Application Rate	Plant Species	AMF Species	AMF Application Rate	Synergistic Effect	Reference
4.38		21.07	1.55	71.2	20	rice straw	10.2	8.7	2.4	3%	*Medicago sativa*	Mixing of four different fungi	3.30%	Y	[105]
7.5	15.69	13.24	0.27	50.23	4	wheat straw	10.2	0.27	0.26	2%	Mulberry	*Glomus intraradices*	2%	Y	[110]
5.91	52	0.0165	1.25		5	tobacco straw	10.3	17.7	4.8	2%	maize	*Glomus versiforme*	5%	Y	[112]
5.91	52	0.0165	1.25		5	tobacco straw	10.3	17.7	4.8	2%	maize	*Funneliformis mosseae*	5%	Y	-
5.91	52	0.0165	1.25		5	tobacco straw	10.3	17.7	4.8	2%	maize	*Rhizophagus intraradices*	5%	Y	-
7.6	17.13	12.62	0.54	46.66	3	wheat straw	10.4	0.59		2%	maize	*Glomus intraradices*	2%	Y	[32]
7.6	17.13	12.62	0.54	46.66	6	wheat straw	10.4	0.59		2%	maize	*Glomus intraradices*	2%	Y	-
7.6	17.13	12.62	0.54	46.66	3	wheat straw	10.4	5.9	0.89	2%	maize	*Glomus intraradices*	2%	Y	[90]
7.6	17.13	12.62	0.54	46.66	6	wheat straw	10.4	5.9	0.89	2%	maize	*Glomus intraradices*	2%	Y	-
6.11	7.14	20.86	1.47	38.46	1	rice straw	10.2	8.7	2.4	5%	*Trifolium*	Mixing two different fungi	6.50%	N	[123]
6.11	37.14	20.86	1.47	38.46	1	rice straw	10.2	8.7	2.4	5%	*Trifolium*	Mixing two different fungi	6.50%	N	-
6.11	107.14	20.86	1.47	38.46	1	rice straw	10.2	8.7	2.4	5%	*Trifolium*	Mixing two different fungi	6.50%	N	-
4.38	36.61	21.07	1.55	71.2	10	rice straw	10.2	8.7	2.4	5%	*Lolium multiflorum*	Mixing of four different fungi	6%	N	[120]
4.38	86.61	21.07	1.55	71.2	10	rice straw	10.2	8.7	2.4	5%	*Lolium multiflorum*	Mixing of four different fungi	6%	N	-
4.38	136.61	21.07	1.55	71.2	10	rice straw	10.2	8.7	2.4	5%	*Lolium multiflorum*	Mixing of four different fungi	6%	N	-
8.27	15	0.0131		50.56	20	Pig manure and bamboo powder mixture				4.50%	Alamo	*Rhizophagus irregularis*	1.10%	N	[124]
8.27	75	0.0131		50.56	20	Pig manure and bamboo powder mixture				4.50%	Alamo	*Rhizophagus irregularis*	1.10%	N	-
7.84	1.45			8	5	reed	8.98	0.0051	0.0023	1%	maize	*Rhizophagus clarus*	1.70%	N	[122]
7.84	1.45			8	10	reed	8.98	0.0051	0.0023	1%	maize	*Rhizophagus clarus*	1.70%	N	-
5.71	26	43.05	3.4	200	3	maize	9.74	8.53	1.21	3%	*Cichorium intybus*	Mixing of four different fungi	10%	Y	[111]

## Data Availability

All used data can be obtained from published articles.

## Supplementary Materials

The following supporting information can be downloaded at: https://www.mdpi.com/article/10.3390/jof10030182/s1, Figure S1: Adapted PRISMA flow diagram, indicating various phases in retrieving published articles from the comprehensive databases to analyze the effectiveness of biochar coupled with arbuscular mycorrhizal fungi in the soil cadmium immobilization.
