# Supplementary material for: A Critical Review of the Effectiveness of Biochar Coupled with Arbuscular Mycorrhizal Fungi in Soil Cadmium Immobilization
Source: J Fungi (Basel). 2024 Feb 28;10(3):182. doi: 10.3390/jof10030182 (PMC10970794; doi:10.3390/jof10030182)
Supplement: Supplementary file 1 [file jof-10-00182-s001.zip › jof-2854348-supplementary.pdf]

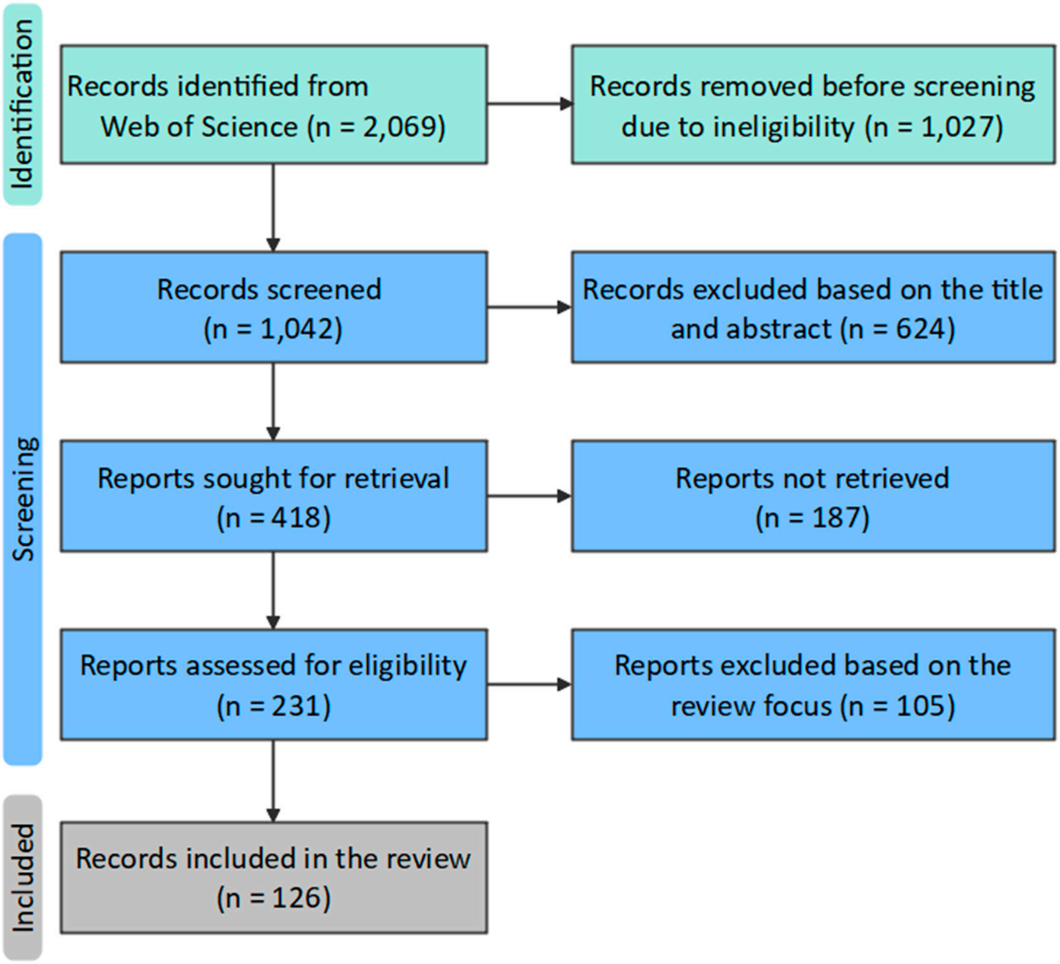

Figure S1. Adapted PRISMA flow diagram, indicating various phases in retrieving published articles from the comprehensive databases to analyze the effectiveness of biochar coupled with arbuscular mycorrhizal fungi in the soil cadmium immobilization.

As detailed in **Figure S1**, each stage of the search and screening process encompassed the inclusion, exclusion, and extraction of pertinent studies. The appropriate articles were exported and stored in CSV format. During the review process, 2,069 articles were identified from Web of Science. Initially, 1,027 ineligible records were excluded from the initial reports. The 1,042 remaining records underwent screening based on the title and abstract level, followed by a full-text assessment. After that, 624 articles were excluded because they did not cover biochar in areas such as remediation/degradation, health, adsorption/sorption, pesticides, waste, and sludge. 418 articles were obtained for in-depth analysis of their full texts. Of these, 187 were excluded because they were preprints, reports, or new papers. Additionally, 105 papers were eliminated from the analysis due to their lack of focus on the review aim. After assessing all exclusion and inclusion criteria, 126 articles were selected for this review paper. The authors scrutinized the records to ensure the reliability of the retrieved sources. The present study employed a systematic search strategy involving searching and screening. The search phase entailed exploring publications directly linked to the investigation of adding biochar-AMF in remediating cadmium-soil contamination through the Web of Science. The search involved meticulously collecting a raw database of target review themes using articles published from 2000-01-01 to 2024-01-01. In this context, the title, abstract, or keywords were used as the search criteria: biochar* and arbuscular mycorrhizal fungi* and soil* and cadmium* and plants* or biochar* and wastes* or sludge*or aqueous solution* with singular and plural. Scholarly articles, chapters from books, and brief communications were considered.
